# Chronic Airways Assessment Test: performance and health predictors in the AlphaNet population

**DOI:** 10.1093/ajrccm/aamag158

**Published:** 2026-03-30

**Authors:** Radmila Choate, Kristen E Holm, Robert A Sandhaus, David M Mannino, Charlie Strange

**Affiliations:** Department of Epidemiology and Environmental Health, University of Kentucky College of Public Health, Lexington, KY, United States; Department of Medicine, National Jewish Health, Denver, CO, United States; AlphaNet, Inc., Coral Gables, FL, United States; Department of Medicine, National Jewish Health, Denver, CO, United States; AlphaNet, Inc., Coral Gables, FL, United States; Division of Pulmonary, Critical Care, and Sleep Medicine, University of Kentucky College of Medicine, Lexington, KY, United States; AlphaNet, Inc., Coral Gables, FL, United States; Division of Pulmonary, Critical Care, Allergy and Sleep Medicine, Medical University of South Carolina, Charleston, SC, United States

*To the Editor:*

The Chronic Airways Assessment Test (CAAT) is a validated, 8-item questionnaire widely used in chronic obstructive pulmonary disease (COPD) to evaluate disease impact on health status.^1^ The CAAT adapts the COPD Assessment Test (CAT) by replacing references to “COPD” with “lung disease” while retaining identical items and scoring. The CAAT is used broadly in both clinical practice and research settings in the COPD and asthma populations^2^; however, its performance in alpha-1 antitrypsin deficiency (AATD)–associated lung disease has not been well studied.^3^^,^^4^ Therefore, this study sought to evaluate the performance of the CAAT in a large cohort of individuals with AATD–associated lung disease and to identify factors associated with worse health status.

## Methods

The study population included individuals with abnormal AAT genotypes prescribed augmentation therapy and enrolled in AlphaNet, a United States–based health management organization for individuals with AATD-associated lung disease. Eligible participants completed the CAAT, provided demographic and health information, and consented to research. The CAAT includes 8 items scored 0 to 5, with a total score of 0 to 40. Breathlessness was assessed using the modified Medical Research Council (mMRC) dyspnea scale. AAT genotype was categorized into 3 groups: the ZZ group, including ZZ, ZNull, and NullNull genotypes; the other genotype group, including SZ, MZ, and other variants; and a missing category for unavailable genotype data.

Descriptive statistics are reported as means with standard deviation (SD) or proportions. Participants were classified as CAAT score <10 (low impact) and CAAT score ≥10 (moderate to very high impact). Between-group differences were evaluated using χ^2^ tests for categorical variables. Standardized differences were calculated to quantify the magnitude of between-group differences independent of sample size,^5^ with thresholds of <0.2 (small), 0.2 to <0.5 (small to moderate), 0.5 to 0.8 (moderate to large), and >0.8 (large). For multicategory variables, the maximum absolute standardized difference was reported.

Candidate predictors for the multivariable analyses included age, sex, AAT genotype category, regular oxygen use, exacerbation frequency, daily productive cough, bronchiectasis, and smoking history. Because of conceptual overlap with CAAT, mMRC was not included. Penalized logistic regression using Least Absolute Shrinkage and Selection Operator (LASSO) was applied to evaluate predictor stability and redundancy among prespecified demographic and clinical variables, with the penalty parameter selected using the Akaike information criterion. Penalized regression retained all prespecified predictors, reflecting their shared but nonredundant contributions. Inference was obtained from a post-LASSO unpenalized logistic regression model including all selected variables. Odds ratios (ORs) with Wald 95% confidence intervals (CIs) are reported.

The study was approved by the University of Kentucky Institutional Review Board (#43435).

## Results

The analytic sample included 779 participants (mean age, 60.2 ± 11.8 years); 59.2% were female, and 41.3% had a ZZ, ZNull, or NullNull genotype. The mean CAAT score was 16.0 ± 10.0, and 55% reported dyspnea with mMRC score ≥2. Overall, 76.8% had CAAT ≥10 (Table 1).

**Table 1:** Comparison of participant characteristics across CAAT score groups.

Characteristic	**Overall (*N*** = **779)**	**CAAT <10 (*n*** = **181 [23.2%])**	**CAAT ≥10 (*n*** = **598 [76.8%])**	*P* value	**Standardized difference** ^a^
**Age, y**				.1440	0.161
** <50**	133 (17.1)	30 (16.6)	103 (17.2)		
** 50-65**	378 (48.5)	78 (43.1)	300 (50.2)		
** >65**	268 (34.4)	73 (40.3)	195 (32.6)		
**Sex**				.0366	0.177
** Female**	461 (59.2)	95 (52.5)	366 (61.2)		
** Male**	318 (40.8)	86 (47.5)	232 (38.8)		
**Regular use of oxygen**				<.0001	0.766
** No**	527 (67.7)	165 (91.2)	362 (60.5)		
** Yes**	252 (32.4)	16 (8.8)	236 (39.5)		
**Exacerbations in the past year**				<.0001	0.822
** 0**	278 (35.7)	108 (59.7)	170 (28.4)		
** 1**	135 (17.3)	39 (21.6)	96 (16.1)		
** ≥2**	366 (47.0)	34 (18.8)	332 (55.5)		
**mMRC dyspnea scale**				<.0001	1.281
** 0**	101 (13.0)	78 (43.1)	23 (3.9)		
** 1**	249 (32.0)	77 (42.5)	172 (28.8)		
** ≥2**	429 (55.0)	26 (14.4)	403 (67.4)		
**Daily productive cough** ^b^				<.0001	0.859
** No**	424 (54.4)	151 (83.4)	273 (45.7)		
** Yes**	355 (45.6)	30 (16.6)	325 (54.4)		
**Bronchiectasis**				.6853	0.035
** No**	722 (92.7)	169 (93.4)	553 (92.5)		
** Yes**	57 (7.3)	12 (6.6)	45 (7.5)		
**Ever smoked**				<.0001	0.393
** No**	250 (32.1)	84 (46.4)	166 (27.8)		
** Yes**	529 (67.9)	97 (53.6)	432 (72.2)		
**Alpha-1 genotype**				.0002	0.410
** ZZ** ^c^	322 (41.3)	99 (54.7)	223 (37.3)		
** Other** ^d^	378 (48.5)	67 (37.0)	311 (52.0)		
** Unavailable**	79 (10.1)	15 (8.3)	64 (10.7)		

Data are expressed as No. (%).

Abbreviations: CAAT, Chronic Airways Assessment Test; mMRC, modified Medical Research Council.

a Standardized difference indicates maximum standardized difference in proportions across categories.

b Question wording: “Over the past 2 years, have you coughed up sputum/mucus from your lungs on a daily basis for at least 3 months each year?”

c ZZ group includes ZZ, ZNull, and NullNull genotypes.

d Other group includes SZ, MZ, and other genotypes.

Compared with the CAAT <10 group, participants with CAAT ≥10 were more likely to report regular oxygen use, ≥2 exacerbations in the prior year, higher mMRC scores, and daily productive cough (*P* <.0001 for all). These variables showed large standardized differences, indicating clinically meaningful between-group differences beyond statistical significance (Table 1). The CAAT <10 group had a higher proportion of males and people who had never smoked, but standardized differences for these comparisons were not large. No significant differences were observed between CAAT groups in age or the presence of bronchiectasis.

**Figure 1:**
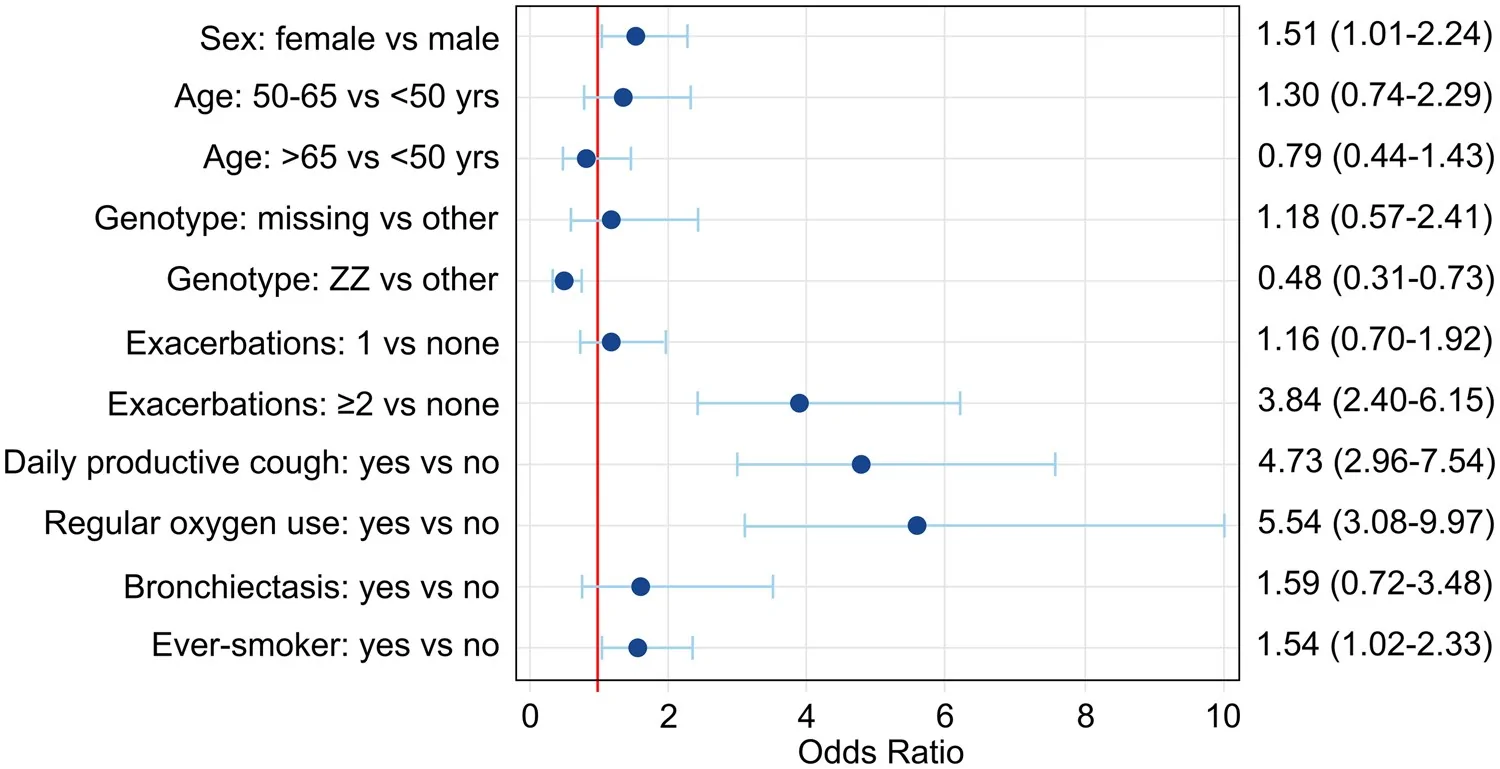
Adjusted odds ratios and 95% confidence intervals for factors associated with Chronic Airways Assessment Test (CAAT) scores of ≥10. Genotype: ZZ group includes ZZ, ZNull, and NullNull; Other group includes MZ, SZ, and other.

In penalized models, all candidate predictors were retained, suggesting limited redundancy among clinically relevant variables. In post-LASSO logistic regression, daily sputum production and oxygen use were the strongest predictors of CAAT ≥10 (ORs of 4.73 and 5.54, respectively) (Figure 1). Exacerbation frequency demonstrated a dose–response relationship, with ≥2 exacerbations associated with CAAT ≥10 (OR, 3.84 [95% CI, 2.40-6.15]). Female sex was independently associated with worse health status compared with males (OR, 1.51 [95% CI, 1.01-2.24]). Individuals with other genotypes, including SZ and MZ, had higher odds of CAAT ≥10 compared with the ZZ genotype group. Smoking history was marginally associated with CAAT ≥10, whereas bronchiectasis and age were retained but not independently associated after adjustment. Model discrimination was strong (c-statistic 0.84).

## Discussion

In this large cohort of individuals with AATD-associated lung disease, over three-quarters reported moderate to very high disease impact as measured by the CAAT. Worse health status was strongly associated with regular oxygen use, exacerbation burden, and daily productive cough, underscoring the central role of symptom burden in patient-reported disease impact. The multivariable model demonstrated good discrimination, supporting the ability of these clinical features to meaningfully differentiate CAAT groups.

Consistent with prior studies in COPD and asthma,^2^ CAAT scores were closely aligned with dyspnea, respiratory symptoms, and exacerbations. Regular oxygen use and sputum production were the strongest predictors of CAAT ≥10, each associated with nearly 5-fold higher odds of worse health status. Exacerbation burden showed a dose-response relationship, with nearly 4-fold higher odds of worse health status among those reporting ≥2 exacerbations, which aligns with prior research in COPD.^6^^,^^7^

Individuals with SZ, MZ, and other genotypes had higher odds of CAAT ≥10 compared with the ZZ genotype group. This may be due to differences such as fewer comorbidities and more beneficial health behaviors among individuals in the ZZ genotype group, which has been demonstrated within AlphaNet. Ever smoking and female sex were independently associated with higher odds of CAAT ≥10, consistent with prior research in AATD and non-AATD cohorts.^4^^,^^8^ Age and bronchiectasis were retained in the model but not independently associated after adjustment, suggesting a limited incremental contribution to CAAT classification.

CAAT provides a brief, low-burden measure^9^ that captures patient-experienced disease impact not reflected by spirometry alone, and parallels associations with clinically important characteristics previously observed in this same population.^10^ Despite growing interest in AATD-specific patient-reported outcomes,^3^ the CAAT remains a practical and informative tool due to its low respondent burden and reliability.

Strengths of this study include the large, well-characterized AlphaNet cohort and standardized CAAT assessment. Limitations include the cross-sectional design and the unavailability of certain clinical measures, such as spirometry. AlphaNet participants are prescribed augmentation therapy and may be more engaged in their health care. Therefore, findings may not generalize to broader or untreated AATD populations outside of structured disease management programs and should be validated in less selected cohorts.

In conclusion, CAAT demonstrated clinically meaningful associations with symptom burden, exacerbations, oxygen use, and selected demographic and behavioral factors in AATD-associated lung disease. These findings support its use as a practical measure of disease impact and a tool for identifying individuals at risk of poor health status in clinical and research settings.

## Supplementary Material

aamag158_Supplementary_Data
